# Stanene: Atomically Thick Free-standing Layer of 2D Hexagonal Tin

**DOI:** 10.1038/srep31073

**Published:** 2016-08-05

**Authors:** Sumit Saxena, Raghvendra Pratap Chaudhary, Shobha Shukla

**Affiliations:** 1 Nanostructures Engineering and Modeling Laboratory, Department of Metallurgical Engineering and Materials Science, Indian Institute of Technology Bombay, Mumbai, MH, 400076 India

## Abstract

Stanene is one of most important of 2D materials due to its potential to demonstrate room temperature topological effects due to opening of spin-orbit gap. In this pursuit we report synthesis and investigation of optical properties of stanene up to few layers, a two-dimensional hexagonal structural analogue of graphene. Atomic scale morphological and elemental characterization using HRTEM equipped with SAED and EDAX detectors confirm the presence of hexagonal lattice of Sn atoms. The position of Raman peak along with the inter-planar ‘d’ spacing obtained from SAED for prepared samples are in good agreement with that obtained from first principles calculations and confirm that the sheets are not (111) α-Sn sheets. Further, the optical signature calculated using density functional theory at ~191 nm and ~233 nm for low buckled stanene are in qualitative agreement with the measured UV-Vis absorption spectrum. AFM measurements suggest interlayer spacing of ~0.33 nm in good agreement with that reported for epitaxial stanene sheets. No traces of oxygen were observed in the EDAX spectrum suggesting the absence of any oxidized phases. This is also confirmed by Raman measurements by comparing with oxidized stanene sheets.

Two dimensional (2D) layered materials have recently gained renewed interest due to their exotic electronic properties along with high specific surface area. The prospects of exploiting these properties in sensing, catalysis, energy storage, protective coatings and electrochromism have witnessed a paradigm shift towards the exploration of these sophisticated 2D materials. The exemplary performance of graphene^1^ which is among the first of these elemental 2D materials have initiated a runaway effect in the pursuit of studying alternative 2D materials. Even though graphene has tunable exotic electronic properties^2^, the spin-orbit (SO) coupling is weak^3^,^4^,^5^ limiting its applications as spin filters, topological insulators etc. Topological insulators by their very nature force the electrons to travel on the surface at very high speeds thereby finding useful applications in electronic and photonic devices. Exploration of group IV elements using first principles calculations have revealed that the SO coupling increases as the atomic weight of the basis atoms in the honeycomb lattice^6^,^7^. Tin is one of the heaviest elements in this series having strong spin-orbit coupling making it a promising applicant for room temperature topological insulator^8^. Thus there is an urgent need to discover novel 2D materials in the post graphene age to overcome its deficiencies.

Here we report the synthesis of few-layer stanene (FLS) using ultra-fast laser-material interactions. FLS is analogous to few-layer graphene and can be visualized by replacing carbon atoms by tin on a graphene lattice. Structural characterization performed using high resolution transmission electron microscopy (HRTEM) equipped with energy dispersive X-ray analysis (EDAX) and selected area electron diffraction (SAED) detectors confirm the presence of hexagonal lattice of Sn atoms. EDAX and comparative Raman studies that oxide phases are absent and rules out the possibility of (111) α-Sn sheets. Height profile measured using atomic force microscopy (AFM) suggests interlayer separation of ~3.3 Å and in good agreement with that of recently reported epitaxial stanene. Further the UV-Vis spectrum and Raman spectrum are in good agreement with the optical spectra and phonon frequencies calculated using first principles techniques. The structural characterization along with optical signature suggests the synthesis of free standing stanene sheets.

## Results and Discussions

Free standing stanene sheets were synthesized by impinging pulses from a tunable Ti:Saphire ultra-fast femto second laser (140 femto-second pulse width and 80 MHz repetition rate) on to a target in liquid medium. This interaction of femo-second laser pulse due to inverse Bremsstrahlung multiphoton absorption process^9^ induce non-equilibrium conditions^10^. This we hypothesize initiates the change of phase from tetragonal structure of the tin target to hexagonal phase of the prepared stanene samples. Even though several studies are available on understanding these complex ablation process at different time scales^11^ undergoing phase transformations^12^ and producing different morphologies^13^, exact reaction process is still not understood and requires further investigations. This process is different from the oxide nano-particle formation using nano-second lasers in which no change of lattice is observed^14^. The laser was tuned at 800 nm focused using a double convex lens with focal length of 20 cm. Target was prepared by cutting a tin ingot (99.98% pure). X-Ray analysis of the target showed that the presence of β phase of tin and the X-Ray data matched exactly with the JCPDS file no. 00-004-0673. The highest intensity peak corresponded to [101] plane. The target placed in a beaker filled with liquid medium and rotated on a motorized turntable. The sample was reduced in a controlled addition of hydrazine to take care of minor oxidation of the sample during synthesis.

**Figure 1:**
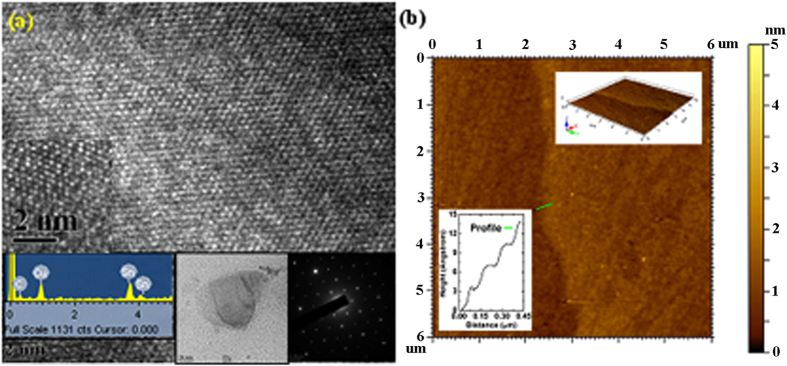
(**a**) HRTEM image of sample showing hexagonal lattice. The inset on the bottom left shows the EDAX spectrum from the same spot. Carbon and copper peaks arises from the TEM grid used. The middle inset shows large area TEM of stanene flake with layers. The inset on the extreme right shows the hexagonal electron diffraction pattern obtained from the sample confirming the presence of hexagonal lattice. (**b**) AFM image showing edge of ~4 layered stanene sheet. The inset on the bottom left shows height profile with several equal steps of height ~3.3 Å suggesting the interlayer separation. The inset at the top right shows a 3D view showing flake like structure.

The samples were characterized for structure, symmetry and chemical composition using HRTEM, equipped with SAED and EDAX detectors. HRTEM image of a representative sample in Fig. 1(a) shows hexagonal arrangement of atoms. This was verified using SAED pattern. The inter-planar ‘d’ spacing calculated using the SAED pattern shown in bottom right inset in Fig. 1(a) was found to be d_1_ ~ 0.25 nm and d_2_ ~ 0.16 nm. These ‘d’ spacing neither agree with that of standard β-tin^15^ and SnO_2_ samples^14^, nor with the ‘d’ spacing obtained from the target obtained using X-Ray diffraction pattern. This suggests structural phase transformation during the synthesis process. These spacing are however in very good agreement with the X-ray diffraction pattern calculated from the optimized low buckled structure obtained using density functional theory (DFT) calculations. These planes are parallel to the zigzag and armchair edges in low buckled stanene lattice respectively. The chemical analysis using EDAX incorporated in TEM shows the peaks corresponding to Sn around ~4 KeV. No signal corresponding to oxygen peaks were observed in the EDAX spectrum in the inset in Fig. 1(a). Sample AFM image in Fig. 1(b) shows the edge of a large stanene flake. The height profile obtained from AFM indicates the presence of 4 layers as shown in bottom left inset in Fig. 1(b). The interlayer spacing was estimated to be ~3.3 Å and is in good agreement with the bi-layer separation obtained using DFT simulations and recently published interlayer spacing in MBE prepared stanene sheets^16^. The inset on the top right in Fig. 1(b) shows 3D view of the AFM image suggests sheet like topology of the sample.

**Figure 2:**
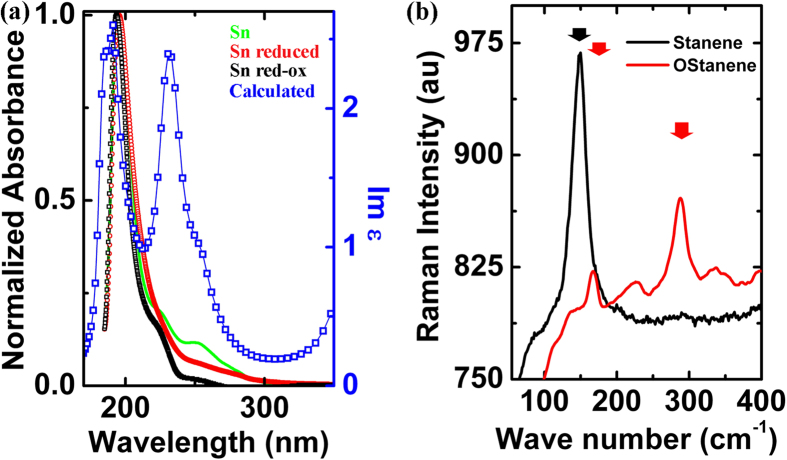
(**a**) Normalized UV spectrum of as prepared stanene sheets (Sn, green), stanene sheets reduced using hydrazine (SnR, red) and subsequently oxidized using H_2_O_2_ (SnRO, black) and ε_2_ component, the imaginary part of the dielectric constant (Calculated, blue) using DFT. (**b**) Micro-Raman spectrum of as prepared stanene and oxidized sample using 532 nm Laser.

The samples were characterized optically using UV-Vis absorption and Raman spectroscopy. The as prepared stanene samples show strong absorption peak ~196 nm (6.325 eV) and two shoulders at ~225 and ~250 nm as seen in Fig. 2(a). The samples were reduced using hydrazine which resulted in disappearance of the shoulder at ~250 nm. However this reappears again on oxidation of the reduced samples using H_2_O_2_. Similar results are observed in the case of graphene^17^. This suggests that the shoulder at ~250 nm appears due to the transitions originate from minor oxidation of the stanene samples. This could possibly arise due to n → π^*^ transitions due to presence of oxygen atoms.

The electronic states near the Dirac point are dominated by π states with major contribution coming from the out of plane p_z_ orbital. This suggests that most of the low energy optical transitions occur due to the transitions between the π and π* states. The σ states lie farther down below the π states about 3 eV below the Fermi level. The optical properties of the material can be described in terms of complex dielectric function ε = ε_1_ + iε_2_ and the absorption coefficient is directly proportional to imaginary part of the complex dielectric function (ε_2_). ε_2_ is obtained using first principles DFT calculations within the generalized gradient approach. Stanene displays pronounced asymmetric peak in the UV region at photon energy of ~6.325 eV. The peak positions at ~191 nm and ~233 nm in the calculated ε_2_ shows a good similarity with the experimental data.

Raman studies were performed using 532 nm continuous wave, 100 mW Nd:YAG fiber coupled laser show the presence of a very strong peak ~149 cm^−1^ (Fig. 2b). This is in good agreement with the frequencies obtained at gamma point for bi-layer low buckled stanene using first principles calculation. This corresponds to the in-plane vibrations of the Sn atoms. The broad shoulder in the range 85 cm^−1^–110 cm^−1^ is understood to arise from out of plane vibrations of the Sn atoms as suggested by selective dynamics of atoms using first principles techniques. It is important to note that no negative phonon frequencies were found at gamma point suggesting the stability of the structure. These Raman frequencies do not correspond to any gamma phonon frequencies reported for (111) α-Sn thin flims, β-tin, SnO or SnO_2_^18^. Prolonged exposure of higher intensity laser beam in air during measurement results in shifting of the spectrum with the emergence of new peak at 288 cm^−1^. This shift is understood to occur due to change in oxidation state of the Sn atoms. The appearance of additional vibrational modes at ~288 cm^−1^ suggest that large does of high intensity laser beam oxidizes stanene layers forming domains of SnO_x_ in stanene layers. The peaks ~288 cm^−1^ are in good agreement with the E_u_ mode observed in the IR spectrum of crystalline SnO_2_^19^.

## Conclusion

In a nutshell we have synthesized stanene up to few layers using ultrafast laser materials interactions. Structural characterizations using HRTEM equipped with EDAX and SAED detectors confirm the hexagonal symmetry of Sn lattice. First principles calculations along with ‘d’ spacing obtained from SAED and XRD measurements corroborate that stanene occurs in low buckled structural form. Optical signature for stanene with buckling height of 0.85 Å obtained using DFT calculations is in very good agreement with that of the UV-Vis absorption spectrum. Absence of oxygen peak in the SAED spectrum suggests absence of any oxidized phase of the material. Comparative Raman spectrum studies of FLS with oxidized samples, Raman peaks reported for (111) α-Sn thin films and the agreement of the frequency in FLS with that of the gamma phonon calculated for bi-layered stanene provide supporting evidence for synthesis of stanene up to few layers.

## Methods

### Experimental

The samples were prepared using tunable Ti: Saphire ultra-fast femto second laser (140 femto-second pulse width and 80 MHz repetition rate) (Coherent) on to a target in hexane. The laser beam was focused using a biconvex lens of focal length 20 cm. The synthesized samples were characterized structurally using HRTEM, SAED, EDAX, (all detectors equipped in Jeol made model: JEM-2100F with EDAX attachment having 0.19 nm point resolution and 200 KV acceleration voltage) AFM (Agilent 5500). Optical characterizations were performed using Raman (Witec Alpha RAS 300) and UV-Vis absorption (Shimadzu UV-2600) spectrometry.

### Computational

First principles were performed using Density functional theory within the generalized gradient approximation (GGA) as implemented in the Vienna ab-initio simulations package (VASP). The structure was first optimized using high energy cutoff of 500 eV. The reciprocal space was sampled using K-Point mesh of 23 × 23 × 1 centered at gamma point. Projector augmented wave (PAW) potentials with exchange correlation as parameterized by Perdew-Burke-Ernzerhof. The unit cell was constructed such that a 2 nm vacuum space was available only along the z direction to avoid any interactions between the two cells in the direction normal to the layers.

## Additional Information

**How to cite this article**: Saxena, S. *et al.* Stanene: Atomically Thick Free-standing Layer of 2D Hexagonal Tin. *Sci. Rep.* **6**, 31073; doi: 10.1038/srep31073 (2016).
